# Supplementary material for: Surgical Outcomes, Long-Term Survivals and Staging Systems of World Health Organization G3 Pancreatic Neuroendocrine Tumors
Source: J Clin Med. 2022 Sep 6;11(18):5253. doi: 10.3390/jcm11185253 (PMC9502090; doi:10.3390/jcm11185253)
Supplement: Supplementary file 1 [file jcm-11-05253-s001.zip › jcm-1884778-supplementary.pdf]

Supplementary file. **Table S1**: The current AJCC 8th TNM staging system and the mTNM staging system for G3 p-NETs.

	**Current AJCC 8th TNM Staging System**	**Proposed mTNM Staging System[A]**
	**T/N/M staging definitions**	
**T1**	Tumors limited to pancreas, 2 cm or less in greatest dimension;	Tumors limited to pancreas, 2 cm or less in greatest dimension;
**T2**	Tumors limited to pancreas more than 2 cm but less than 4 cm in greatest dimension;	Tumors limited to pancreas more than 2 cm but less than 4 cm in greatest dimension;
**T3**	Tumors limited to pancreas, more than 4 cm in greatest dimension or tumors invading duodenum or bile duct;	Tumors limited to pancreas, more than 4 cm in greatest dimension or tumors invading duodenum or bile duct;
**T4**	Tumor invading adjacent organs (stomach, spleen, colon, adrenal gland) or the wall of large vessels (celiac axis or the superior mesenteric artery).	Tumor invading adjacent organs (stomach, spleen, colon, adrenal gland) or the wall of large vessels (celiac axis or the superior mesenteric artery).
**N0**	No regional lymph node metastasis;	No regional lymph node metastasis;
**N1**	Regional lymph node metastasis.	1-3 regional lymph node metastasis;
**N2**	NA.	≧4 regional lymph node metastasis.
**M0**	No distant metastasis;	No distant metastasis;
**M1**	Distant metastasis.	Distant metastasis.
	**Clinical staging definitions**	
**Stage I**	T1 N0 M0;	**A**: T1 N0 M0;<br>**B**: T2 N0 M0;
**Stage II**	**A**: T2 N0 M0;<br>**B**: T3 N0 M0;	**A**: T3 N0 M0;<br>**B**: T1-3 N1 M0;
**Stage III**	**A**: T4 N0 M0;<br>**B**: Any T N1 M0;	Any T N2 M0, T4 Any N M0;
**Stage IV**	Any T Any N M1.	Any T Any N M1.

Abbreviations: AJCC: American Joint Committee on Cancer; TNM: tumor-node-metastasis; p-NETs: pancreatic neuroendocrine tumors; mTNM: modified tumor-node-metastasis; G: grading; T: primary tumor; N: regional lymph node; M: distant metastasis; NA: not applicable.

A: The mTNM staging system was originally proposed by Zhang et al. [21].
